# *TTC7A-ALK*, a novel *ALK* fusion variant identified in a patient with metastatic lung adenocarcinoma, exhibits excellent response to crizotinib

**DOI:** 10.1016/j.tranon.2025.102345

**Published:** 2025-03-06

**Authors:** Meijin Huang, Xiangqing Zhu, Wenmang Xu, Jun Zhu, Xin Xun, Bin Su, Hong Chen

**Affiliations:** a Department of Oncology, 920th Hospital of Joint Logistics Support Force, PLA, Yunnan, China; b Department of Basic Medical Laboratory, 920th Hospital of Joint Logistics Support Force, PLA, Yunnan, China; c Department of Pathology, 920th Hospital of Joint Logistics Support Force, PLA, Yunnan, China

**Keywords:** *TTC7A-ALK fusion*, Next generation sequencing, Targeted therapy, *ALK inhibitor*, Non-small cell lung cancer

## Abstract

Non-small cell lung cancer (NSCLC) remains a leading cause of cancer-related mortality worldwide. *ALK* gene rearrangement has been identified in 3 % to 5 % of the patients with NSCLC. Thanks to the advancements in second-generation sequencing technology, an increasing number of novel fusion partners have been identified. In our research, we discovered a rare *ALK* fusion, *TTC7A-ALK*, in a patient with advanced lung adenocarcinoma using targeted next-generation sequencing (NGS). After being diagnosed with advanced lung adenocarcinoma with *TTC7A-ALK* fusion*,* the patient received crizotinib treatment and achieved a progression-free survival of 29 months. Additonanlly, we conducted further functional analyses on this fusion protein to assess its oncogenic potential. Similar to *EML4-ALK*, the *TTC7A-ALK* fusion protein can promote the growth of Ba/F3 cells under IL-3-independent conditions in vitro. In vivo studies demonstrate that the *TTC7A-ALK* fusion protein could enhance the tumorigenesis of NIH3T3 cells in nude mice, which can be suppressed by crizotinib. Mechanistic studies indicated that the ectopic expression of *TTC7A-ALK* in 293T cells led to the hyperactivation of downstream MAPK and PI3K/Akt pathways, which can be inhibited by crizotinib. Furthermore, upon tumor progression, the patient transitioned to alectinib, which provided rapid symptom relief and controlled the majority of lesions. Conclusionly, we identified and validated *TTC7A-ALK* as a oncogenic fusion in NSCLC. The patient demonstrated a significant clinical benefit from sequential treatment with crizotinib and alectinib, highlighting *TTC7A-ALK* as a novel therapeutic target for *ALK* inhibitors. These findings extend the spectrum of actionable *ALK* fusions and promote the inclusion of rare fusion detection in clinical diagnostic processes and treatment strategies.

## Highlights

• We first identified a rare fusion form of *ALK* rearrangements (*TTC7A–ALK*) in an advance NSCLC patient by targeted next generation sequencing.
• Our study provides valuable information on the response of NSCLC patients with *TTC7A-ALK* fusion to crizotinib.
• We constructed the stable expression of *TTC7A-ALK* in three non-cancer cell lines and demonstrated the involvement of this fusion protein in tumorgenesis.
• Signaling pathway studies suggested that *TTC7A-ALK* induces overactivation of MAPK and PI3K/AKT pathways.
• When the patient experienced resistance to crizotinib, alectinib immediately relieved the patient's symptoms and controlled most of the lesions, suggesting that the relapsed tumor is still *ALK*-driven.

## Introduction

Lung cancer (LC) is currently the leading cause of cancer-related mortality worldwide, representing a significant global health challenge [1]. Histologically, LC is classified into two major types: small-cell lung carcinoma (SCLC), which accounts for approximately 15 % of cases, and non-small-cell lung carcinoma (NSCLC), which constitutes the remaining 85 % [[2], [3], [4]]. The prognosis for patients with advanced NSCLC remains poor, with a 5-year overall survival rate of <5 % [5]. Over the past 30 years, targeted therapies specific to driver genes involved in the development of NSCLC have significantly extended the survival of patients with advanced NSCLC who are positive for these driver genes. The molecular landscape of NSCLC has unveiled a complex network of genetic alterations that play pivotal roles in tumorigenesis and therapeutic responsiveness. The most common driver gene mutations in NSCLC are *epidermal growth factor receptor (EGFR)* gene mutations and *anaplastic lymphoma kinase (ALK)* fusion gene positivity. Recently, in addition to *EGFR* and *ALK*, researchers have discovered an increasing number of driver genes in NSCLC, but these genes typically have a mutation rate of <5 % in NSCLC and are referred to as uncommon mutations. Examples include *ROS proto-oncogene 1 (ROS1), B-raf proto-oncoprotein (BRAF) V600E, neurotrophic tropomyosin receptor kinase (NTRK1), human epidermal growth factor receptor (HER2), mesenchymal-to-epithelial transition (MET), rearranged during transfection (RET), EGFR exon 20 insertions*, and *kirsten rat sarcoma viral oncogene (KRAS) G12C mutations*. Targeted drugs for these uncommon mutations in NSCLC have been increasingly developed and applied in clinical practice, achieving promising results [6].

*ALK* fusion genes play a crucial role as driver genes in non-small cell lung cancer (NSCLC). Detecting these fusions, which occur in approximately 3 %−7 % of NSCLC cases, is vital for tumor diagnosis, prognosis assessment, and targeted therapy., particularly for adenocarcinoma subtypes [7]. *Echinoderm microtubule associated protein like 4 gene (EML4)* is the most common partner for *ALK* fusion [[7], [8], [9], [10]]. Methods for detecting *ALK*-positive NSCLC have evolved from fluorescence in situ hybridization and immunohistochemistry to next-generation DNA sequencing, targeted RNA sequencing, and whole transcriptome sequencing. Consequently, these deep sequencing methods have led to the identification of a greater variety of fusion partners in *ALK*-positive NSCLC [[11], [12], [13], [14], [15], [16], [17], [18], [19]]. Sai-Hong Ignatius Ou et al., have compiled a list of the *ALK* fusion partners including intergenic rearrangements. By the end of January 2020, a total of 90 distinct *ALK* fusion partners (including noncoding RNAs) have been identified in the literature [20]. Additionally, we extensively searched PubMed publications, conference/assembly abstracts, and presentations to identify new *ALK* fusion partners since 2020, including non-coding RNA. Overall, as of January 2025, including the fusions we discovered, a total of 51 different *ALK* fusion partners have been identified in the literature [[21], [22], [23], [24], [25], [26], [27], [28], [29], [30], [31], [32], [33], [34], [35], [36], [37], [38], [39], [40], [41], [42], [43], [44], [45], [46], [47], [48], [49], [50], [51], [52], [53], [54], [55], [56]] (Table1).

**Table 1:** Catalog fo fusion partners in *ALK*-positive NSCLC since 2020.

No.	Fusion Partner	Year Published in Print/Presented	Response to ALK TKI at the Time of Publication	Tumor Source	Method of Detection	Variant Frequency	FISH/ IHC	References
1	*LOC101927285*	2020	PR to crizotinib	Tumor	NGS	22.92 %	ND/+	Zhao [21]
2	*COX7A2L*	2020	PR to crizotinib	Tumor	NGS	41.2 %	ND/+	Cai [22]
3	*LINC01210*	2020	PR to crizotinib	Tumor	NGS	34.7 %	ND/+	Cai, 2020[22]
4	*ATP13A4*	2020	PR to crizotinib	Tumor	NGS	21.5 %	ND/+	Cai [22]
5	*TPM3*	2020	PR to crizotinib	Tumor	NGS	21.76 %	ND/+	Zhao [23]
6	*MRPL13*	2020	PR to crizotinib	plasma circulating tumor DNA	NGS	5.73 %	ND/+	Jiao [24]
7	*PPP1CB*	2020	PR to crizotinib	plasma circulating tumor DNA	NGS	12.80 %	ND/+	Jiao [24]
8	*ATIC*	2020	PR to crizotinib	Tumor	NGS	16.33 %	+/+	Wu [25]
9	*CCNY*	2020	PR to crizotinib	Tumor	NGS	16.64 %	+/+	Wu [25]
10	*PDK1*	2021	PR to alectinib	Tumor	NGS	12.85 %	ND/+	Zeng [26]
11	*STK3*	2021	PR to crizotinib	Tumor	NGS	3.1 %	ND/+	Feng [27]
12	*MRPS9*	2021	PR to alectinib	Tumor	NGS	NR	ND/ND	Zhou [28]
13	*PTH2R*	2021	PR to ceritinib	Tumor	NGS	NR	+/ND	Shen [29]
14	*DCTN1*	2021	PR to alectinib	plasma circulating tumor DNA	NGS	0.23 %	+/+	Yin [30]
15	*CCDC85A*	2022	PR to alectinib	Tumor, blood specimen	NGS	NR	ND/+	Lin [31]
16	*SLC8A1/LINC01913*	2022	PR to crizotinib	Tumor	NGS	NR	+/+	Wang [32]
17	*IGR (upstream C2orf16)*	2022	PR to alectinib	Tumor	NGS	22.6 %	ND/+	Liao [33]
18	*IGR(downstream ZIC4)*	2022	Resistant to alectinib	Tumor	NGS	9.7 %	ND/+	Liao [33]
19	*ARID2*	2022	PR to crizotinib	Tumor	NGS	1.67 %	ND/+	Tao [34]
20	*SSH2*	2022	PR to crizotinib	Tumor	NGS	0.85 %	ND/+	Tao [34]
21	*LTBP1*	2022	PR to alectinib	Tumor	NGS	10.28 %	+/+	Li [35]
22	*LMO7*	2022	PR to crizotinib	Tumor	NGS	NR	ND/ND	Yang [36]
23	*HLA-DRB1*	2022	PR to crizotinib	The serum sample	NGS	NR	ND/ND	Gao [37]
24	*GCA*	2022	PR to alectinib	Tumor	NGS	17.18 %	+/+	Zhai [38]
25	*HIVEP1*	2022	PR to alectinib	Tumor	NGS	7.6 %	+/+	Gu [39]
26	*CTNND1*	2022	PR to alectinib	Tumor	NGS	NR	ND/+	Tian [40]
27	*CPE*	2022	PR to alectinib	Tumor	NGS	NR	ND/+	Qin [41]
28	*CLHC1/RNT4*	2022	PR to crizotinib	Tumor	NGS	39.97 %	+/+	Xia [42]
29	*CLIP1*	2022	PR to alectinib	pleural effusion sample	NGS	0.9 %	+/+	Yuan [43]
30	*SETD3*	2023	PR to crizotinib	Tumor	NGS	36.0288 %	ND/+	Dai [44]
31	*PPFIA1*	2023	PR to alectinib	Tumor	NGS	10 %	ND/+	Yan [45]
32	*C2orf91(intergenic)*	2023	PR to alectinib	Tumor	NGS	13 %	ND/+	Yan [45]
33	*RNF144A*	2023	PR to crizotinib	Tumor	NGS	36.05 %	ND/+	Li [46]
34	*SETD2*	2023	PR to alectinib	Tumor	NGS	25.20 %	ND/+	Zhu [47]
35	*LIMS1*	2023	No response to crizotinib	Tumor	NGS	12.1 %	ND/+	Shi [48]
36	*LDLR*	2023	PR to crizotinib	Tumor	NGS	NR	ND/ND	Shu [49]
37	*DAB1*	2024	NR	Tumor	NGS	NR	ND/+	Xia [50]
38	*KCMF1*	2024	NR	Tumor	NGS	NR	ND/+	Xia [50]
39	*KIF13A*	2024	NR	Tumor	NGS	NR	ND/+	Xia [50]
40	*LOC643770*	2024	NR	Tumor	NGS	NR	ND/+	Xia [50]
41	*XDH*	2024	NR	Tumor	NGS	NR	ND/+	Xia [50]
42	*PLEKHA7*	2024	PR to alectinib	pleural effusion sample	NGS	16.60 %	ND/+	Li [51]
43	*INPP5D*	2024	PR to alectinib	pleural effusion sample	NGS	13.92 %	ND/+	Li [51]
44	*SQSTM1*	2024	PR to ensartinib	Tumor	NGS	NR	ND/+	Wang [52]
45	*LOC399815*	2024	PR to alectinib	The peripheral blood	NGS	8.16 %	ND/+	Li [53]
46	*PLEKHA7*	2024	PR to alectinib	pleural effusion sample	NGS	16.60 %	ND/ND	Li [53]
47	*INPP5D*	2024	PR to alectinib	pleural effusion sample	NGS	13.92 %	ND/ND	Li [53]
48	*UGP2*	2024	PR to alectinib	Tumor	NGS	NR	ND/+	Chen [54]
49	*SV2B*	2024	PR to alectinib	Tumor	NGS	41.01 %	+/+	Chen [55]
50	*SQSTM1*	2024	PR to alectinib	Tumor	NGS	NR	ND/+	Brenda Paola Rodriguez Arroyo [56]
51	*TTC7A*	2025	PR to crizotinib	The peripheral blood	NGS	9 %	ND/+	

In our study, the patient is a case of primary lung cancer with multiple metastases to the liver, pancreas, kidneys, bone, and other sites. We performed a bronchoscopic biopsy to complete the pathological examination and confirm the diagnosis. To enhance the accuracy and reproducibility of detection, which in turn better guides clinical treatment and advances related research, we adhered to the guidelines for detecting novel *ALK* fusions [57]. We selected blood samples, which underwent standardized processing and were subsequently analyzed using high-throughput sequencing. Additionally, we employed immunohistochemistry (IHC) as a complementary technique to assess the expression of fusion proteins. Consequently, we identified a novel *TTC7A* (*Tetratricopeptide repeat domain-7A*)-*AL*K fusion event and meticulously characterized them, detailing the fusion partner genes, fusion sites, and fusion types (e.g., head-to-head, tail-to-tail fusions). Upon conducting our research, we found that this particular fusion has been documented for the first time. To date, there have been no reports of the identical fusion protein occurring in the primary tumor. Additionally, there is currently a lack of data regarding the treatment of such fusions with crizotinib and alectinib. If possible, it would be beneficial to examine the gene expression of both the primary tumor and metastatic foci. We further explored the biological significance and molecular mechanisms of these new fusions. For instance, the novel fusion might exhibit enhanced oncogenic potential or heightened sensitivity to specific drugs, thus presenting new targets for personalized treatment strategies. Our findings have significantly contributed to the guidelines for detecting novel *ALK* fusions. They have expanded the database of known fusion events, offered fresh insights into fusion event research, and provided valuable data and experience for refining and updating the guidelines.

**Fig. 1:**
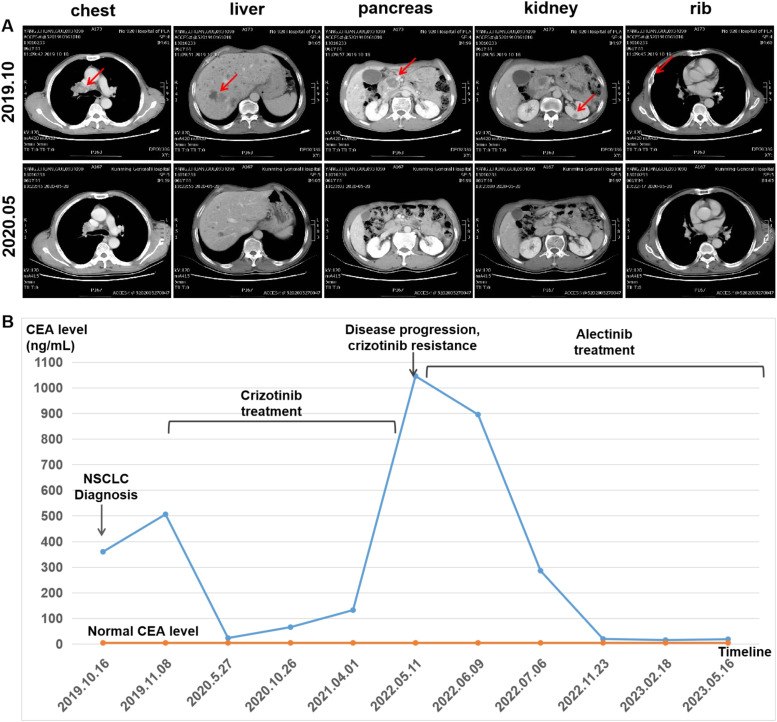
Clinical diagnostic results of a patient with NSCLC. (A) Radiological features before and after therapy. Upper panel: CT scans taken in October 2019 at baseline showed a primary lung lesion in the upper right hilar area, along with metastases to mediastinal lymph node, ipsilateral lung, liver, pancreatic head, kidney and right rib. Lower panel: Partial response (PR) assessment during crizotinib treatment demonstrated significant shrinking of lung lesions and elimination of metastases in May 2020. (B) The chart showed the changes in carcinoembryonic antigen (CEA) levels in the patient from diagnosis to the treatment with crizotinib.. The normal CEA level and checkpoints of the treatment history are labeled in the chart.

Although five *ALK* tyrosine kinase inhibitors (TKIs) have been approved for the treatment of *ALK*-positive non-small cell lung cancer, the sensitivity of these newly discovered rare *ALK* fusion variants to *ALK* TKIs remains unclear. Among these therapeutic agents, crizotinib, a small-molecule tyrosine-kinase inhibitor that targets *ALK, ROS1*, and *MET*, was the first to be approved by the US Food and Drug Administration in 2011 for first-line treatment of metastatic NSCLC with *ALK* rearrangements [58]. However, the response of different *ALK*-positive NSCLC patients to *ALK*-TKIs is heterogeneous. Some patients can benefit greatly from *ALK*-TKI treatment, while others have little or no effect. With the advent of the era of precision treatment, it is very important to clarify the clinical significance of these atypical variants. In terms of treatment response, comparative imaging studies before and after treatment showed that the metastatic lesions had a better response to crizotinib (see Fig. 1). These findings suggest that in this case, compared to the primary tumor, the metastatic lesions showed a more pronounced response to crizotinib treatments. The results of this study have important implications for clinical treatment, such as providing new therapeutic targets and drug options for patients with metastatic *ALK*-positive tumors.

In the future, we will further explore the dynamic changes of fusion proteins at different stages of tumor progression and develop more effective drug combination treatment strategies.

## Materials and methods

### Clinical sample collection

A 61-year-old man with a history of smoking for 20 years was admitted to the Oncology Department of our hospital in August 2019 due to chest pain on his right side accompanied by weight loss for more than a month. The patient has given written consent for specimen collection and genetic testing. The study was reviewed and approved by the Medical Ethics Committee of 920th Hospital of People's Liberation Army (PLA) Joint Logistics Support Force and was conducted in compliance with relevant laws, the Declaration of Helsinki, and other ethical principle (IRB NO.2022–027–01.). We gathered clinical data utilizing the medical records information system by our hospital. The data included demographic information such as gender, age, pathological diagnosis, tumor differentiation degree, tumor location, tumor stage, CA19–9, CA125, CEA levels at the time of diagnosis and treatment history. The TNM staging of LC adhered to the AJCC 8th Edition standard.

Formalin fixed and paraffin embedded (FFPE) sections, obtained via electronic bronchoscopic tissue biopsy at the time of diagnosis, were used for immunohistochemistry to confirm the pathological diagnosis. After pathological confirmation, peripheral blood of the patient was analyzed using targeted next-generation DNA sequencing (NGS) with NGS-panel 16 assay (Jiaxin Yunying Pharmaceutical, Jiangsu, China). During crizotinib treatment, 10 ml of peripheral blood was collected by EDTA-coated tubes (BD Biosciences, Mississauga, ON) at each follow-up visit. These blood samples were used to monitor the expression levels of tumor markers, providing valuable insights into the patient' s response to treatment and disease progression.

### DNA extraction and library construction

The tumor DNA was extracted using a human tissue DNA extraction kit (YunYing Medical Technology Co. Ltd.) according to the manufacturer's protocols. DNA was eluted in the elution buffer, and concentration and purity were assessed using a NanoDrop spectrophotometer. DNA was stored at-20 °C until use. Library preparation was constructed using the VAHTS Universal DNA Library Prep kit for Illumina® sequencing (Illumina, Inc.). Target enrichment was performed using optimized probes (YunYing Medical Technology Co. Ltd.) that targeted the exons of 16 lung cancer-associated genes and specific introns. Sequencing was performed on an Illumina®NextSeq500 platform (Illumina, Inc.) according to the manufacturer's protocols.

### Next-generation sequencing (NGS)- based assay

The Fastqc software (version 0.11.2; http://www.bioinformatics.babraham.ac.uk/projects/fastqc) and customized python scripts were used for screening FASTQ files, and the adaptor sequences and sequences with Q < 30 were removed.Clean reads were mapped to the reference human genome GRCh37-hg19 using Burrows-Wheeler Aligner version 0.7.7. (https://github.com/lh3/bwa).Bam files were then realigned and recalled using GenomeAnalysisTK version 3.5 (https://software.broadinstitute.org/gatk/), which was also used to detect mutations. Somatic mutations with ≥2 % mutant allele frequency, and with at least 20 supporting reads were detected using VarScan version 2.3.2 (http://varscan.sourceforge.net/).

Pindel version 0.2.5b8 (https://www.sanger.ac.uk/science/tools/pindel) was used for indel detection using default parameters. Structure variation was identified using FACTERA version 1.4.4 with default parameters (https://factera.stanford.edu/).

Copy number variations were detected using ONCOCNV version 6.4 with default parameters (http://boevalab.inf.ethz.ch/ONCOCNV/).

The amplification refractory mutation system (ARMS)-PCR was performed to detect all mutations with an allele frequency between 1 and 10 %. All statistical analyses were performed in R (version 1.8.1) [59] and RStudio (version 0.99.903) [60].

### Cell culture and expression constructs

NIH3T3 (RRID: CVCL_0594), HEK-293T (RRID: CVCL_0063), Ba/F3 (RRID: CVCL_0161) cell lines were obtained from American Type Culture Collection (ATCC) and cultured at 37 °Cin a humidified atmosphere with 5 % CO_2_ for routine maintenance and all experiments unless noted otherwise. NIH3T3 cells and HEK-293T cells were maintained in Dulbecco's Modified Eagle's Medium (DMEM; Life Technologies) supplemented with 10 % fetal bovine serum (FBS) and 1 % Penicillin/Streptomycin (Wisent Inc., Quebec, CA). Ba/F3 cells were cultured in RPMI-1640 (Life Technologies) supplemented with 10 % FBS, 1 % Penicillin/Streptomycin, and 10 ng/ml mouse interleukin-3 (IL-3; Cell Signaling Technology, Danvers, MA)

The human *EML4-ALK(V1)* cDNA and *TTC7A-ALK* cDNA were synthesized based on previously published sequences [22] and subcloned into the pEZ-Lv201 retroviral vector (GeneCopoeia). The user manual of the vector have been uploaded as supplementary materials. To generate retroviruses, 1 × 10^6^ HEK-293T packaging cells were cotransfected with 3 μg pEZ-Lv201-puro retroviral and 3 μg EcoPac packaging plasmids using FuGENE HD Transfection Reagent (Promega, Madison, WI). After overnight incubation, the transfection media was replaced with fresh growth medium. Virus-containing supernatants were collected at 48 h post-transfection, and filtered through a 0.45 μm filter to remove cell debris. To establish stable cell lines, Ba/F3 cells and NIH3T3 cells were transduced in 6-cm^2^ cell culture plates with 2 ml virus-containing medium in the presence of 8 μg/ml polybrene. The transduction was carried out for 24 h, followed by selection with 2 μg/mL puromycin for 48–72 h. After puromycin selection, transduced cells were re-plated and expanded for subsequent experiments.

### Cell proliferation assays

Cell proliferation was assessed using Ba/F3 cell lines stably expressing *EML4-ALK(V1), TTC7A-ALK*, wild-type *ALK*, empty pEZ-Lv201 control vector and a blank control. cell were seeded at a density of 0.2 × 10^4^ cells per well in a 96-well plate containing interleukin-3 (IL-3)-free medium and treated with crizotinib at various concentrations for 96 h. After incubation, Alamar Blue reagent (Life Technologies) was added to each well and incubated for an additional 4 h. Cell viability was then determined by measuring absorbance at 450 nm using a Synergy H1 microplate reader (BioTek, Winooski, VT). The data were analyzed to evaluate the effects of crizotinib on cell proliferation under the specified experimental conditions.

### Mouse xenograft studies

All animal experimental protocals were approved by the Ethics Committee of 920th Hospital of People's Liberation Army (PLA) Joint Logistics Support Force and followed the guidelines of laboratory animal care in the Declaration of Helsinki. Male nu/nu mice (6 weeks old) were obtained from SPF Biotech (SPF (Beijing) Biotechnology Co., Ltd.). The certificate of quality for experimental animals was uploaded in the attachment. Animals were randomized into four groups (NIH3T3; NIH3T3+nc; NIH3T3+*TTC7A-ALK*; NIH3T3+Lv-*EML4(V1)-ALK*), with six samples per group. NIH3T3 cells (1 × 10^6^) harboring the indicated expression vectors were resuspended in Matrigel (BD Biosciences) and injected subcutaneously in 200 μL into the right flank of 6-week-old athymic nude mice [61], and allowed to grow 2 weeks before the administration of crizotinib. Two weeks later, mice were dosed with crizotinib (Cell Signaling Technology) (0.6 mg/day) once daily for 2 weeks. The tumor volumes were monitored three times weekly using the formula= (width^2^ × length)/2. Mice were sacrificed 2 weeks after crizotinib treatment. Efforts were made to ensure the animals suffered minimally.

### Western blotting assay

Total protein was extracted using a protein extraction reagent (Roche, Switzerland), and quantified with a BCA protein assay kit (Pierce Biotechnology, USA). Equal amounts of the protein (40 µg) were separated by 12–15 % SDS-polyacrylamide gels and transferred onto nitrocellulose membranes. Then, the membranes were blocked with 5 % fat-free milk in TBST (Tris-buffered saline containing 0.1 % Tween 20) at room temperature for 1 h, and subsequently incubated with the primary antibody at 4 °C overnight. Anti-Akt (Cell Signaling Technology, Cat# 9272, RRID: AB_329,827), anti-phospho-Akt (Cell Signaling Technology, Cat# 13,038, RRID: AB_2,629,447), anti-ERK (Cell Signaling Technology, Cat#4695, RRID: AB_390,779), anti-phospho-ERK (Cell Signaling Technology Cat# 4370, RRID: AB_2,315,112) were purchased from Cell Signaling Technology (1:1000); Anti-TTC7A antibody (Abcam, Cat# ab129448, RRID: AB_11,157,386), anti-EML4 antibody (Abcam, Cat# ab85834, RRID: AB_1,924,957) was obtained from Abcam (1:1000); β-actin antibody (Santa Cruz Biotechnology, Cat# sc-47,778, RRID: AB_626,632) as an internal control was obtained from Santa Cruz Technology (1:10,000). After incubation with goat anti-mouse (Abcam, Cat#ab205719, RRID: AB_2,755,049) or anti-rabbit IgG (Abcam, Cat#ab205718, RRID:AB_2,819,160) at 37 °C for 1 hour, protein bands were visualized using the ECL chemiluminescence detection kit (Pierce Biotechnology, USA).

### Statistical analysis

All experiment was conducted with three biological replicates as controls, and each experiment was performed at least three times. Data was presented as mean ± standard deviation (SD). Statistical analyses and graphical representations were performed using GraphPad Prism™ software. A p-value < 0.05 was as the threshold for statistical significance. This approach ensured reproducibility and robustness in the interpretation of experimental results.

## Results

### Detection of a novel *TTC7A-ALK* fusion in a NSCLC patient

**Fig. 2:**
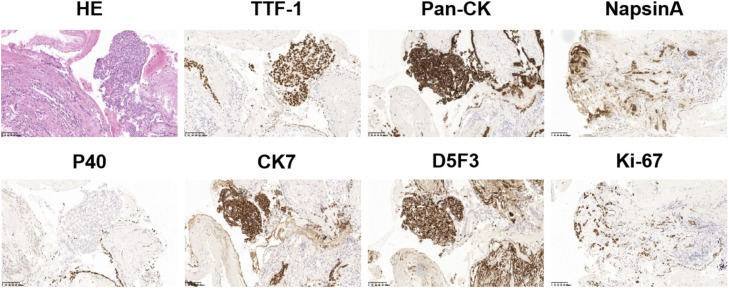
Pathological examination of the patient. Hematoxylin and eosin (H&E) staining of the biopsy specimen showed poorly differentiated NSCLC. IHC showed positive expression for TTF-1, pan-CK, NapsinA, CK7, D5F3 (*ALK* protein), and Ki-67, and negative expression for P40, revealing an adenocarcinoma of lung origin. Images were captured at 200 × magnification.

**Fig. 3:**
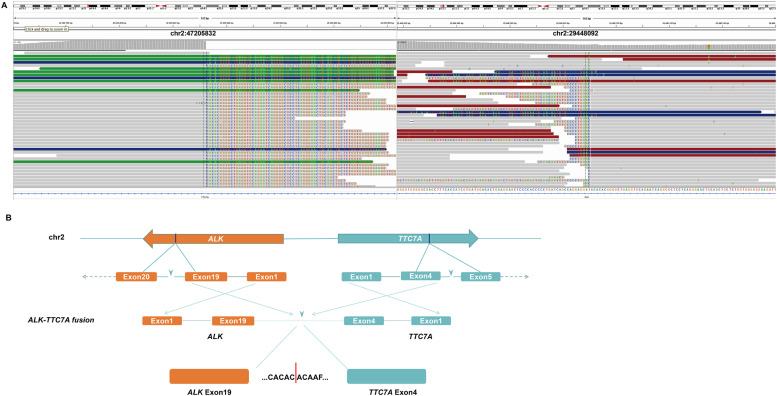
Identification and validation of the *TTC7A-ALK* fusion in a lung cancer patient. (A) Sequencing reads of *ALK* and *TTC7A* were visualized by the Integrative Genomics Viewer (IGV), confirming the presence of a *TTC7A-ALK* fusion. (B) A schematic map showing the structure of the *TTC7A-ALK* fusion locus. The fusion involves exons 1–4 of *TTC7A* (blue) fused to exons 1–19 of *ALK* (orange) via the junction of exon 4 of *TTC7A* and exon 19 of *ALK*.

A 61-year-old man with a history of smoking for 20 years admitted to our hospital in August 2019 with a chief complaint of right-sided chest pain and weight loss persisting for 1 month. A lung computed tomography (CT) scan revealed a mass in the upper right hilar area measuring 3.6 cm × 3.1 cm, along with enlarged mediastinal lymph nodes (the largest lesion measuring) and bone destruction in the right rib area and uneven bone density in the thoracic vertebrae. An abdominal computed tomography (CT) scan displayed multiple metastatic lesions in the liver, pancreatic head and kidney (Fig. 1A). The patient' s serum carcinoembryonic antigen (CEA) level was markedly elevated at 360.43 ng/mL 360.43 ng/mL (normal range: <5 ng/mL) (Fig. 1B). Then, the patient underwent an electronic bronchoscopy biopsy for pathological evaluation. Hematoxylin and eosin (H&E) staining demonstrated a typical adenocarcinoma with a poorly differentiated morphology. Immunohistochemical (IHC) analysis showed positivity for pan-CK, TTF-1, CK7, and NapsinA, and negativity for P40 and P63, consistent with adenocarcinoma. Additionally, IHC staining using the D5F3 clone (Ventana, Oro Valley, Arizona) showed diffuse cytoplasmic expression of *ALK* in the cytoplasm of tumor cells (Fig. 2). Based on the clinical, radiographical and pathological findings, the patient was diagnosed with right hilum lung adenocarcinoma at stage IVB (T4N2M1c). According to the 2019 NCCN guidelines for non-small cell lung cancer, in the stratification of treatment for patients with advanced non-small cell lung cancer at initial diagnosis, the patient should undergo genetic testing and PD-L1 testing. However, for several reasons, PD-L1 testing was not performed. Subsequently, peripheral blood of the patient was subjected to targeted next-generation DNA sequencing (NGS) using the NGS-panel 16 (Jiaxin Yunying Pharmaceutical, Jiangsu, China). Mutation profiling identified a rearrangement on chromosome 2 with a mutant allele frequency (MAF) of 9 %, resulting from an inversion between exon 4 of *TTC7A* (2q12.3) and exon 19 of *ALK* (2p23) (Fig. 3).

### *TTC7A-ALK* fusion induces IL-3-independent cell growth and can be suppressed by crizotinib

**Fig. 4:**
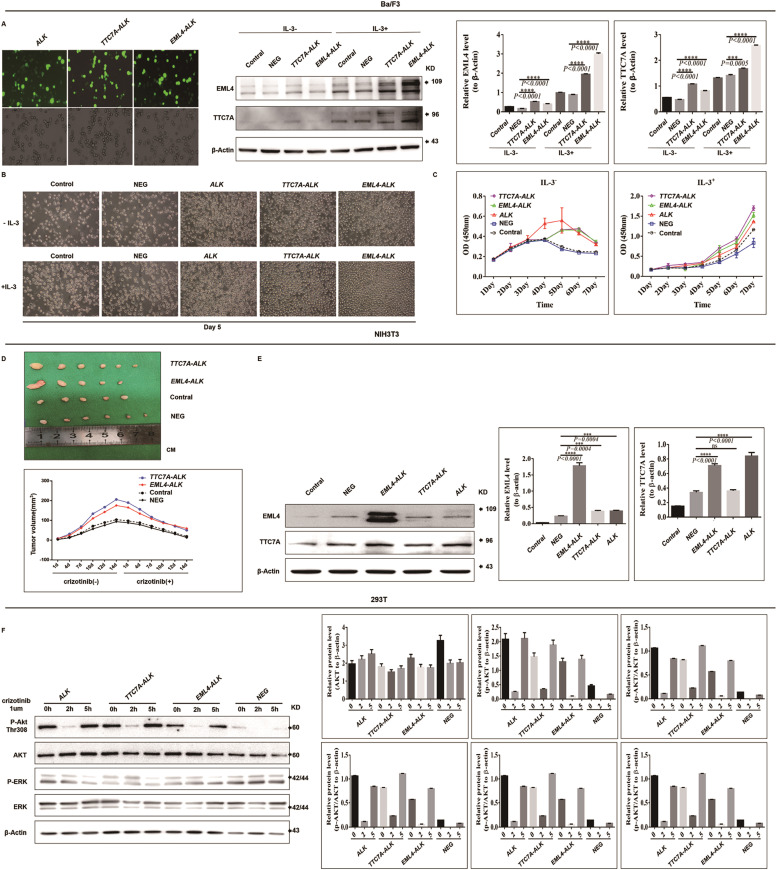
Transforming activity of *TTC7A-ALK* and its response to crizotinib treatment. (A) Fluorescence images of Ba/F3 cells transfected with LV-*ALK*, LV-*TTC7A-ALK*, or LV-*EML4-ALK* constructs, along with western blot and quantification analysis of EML4 and TTC7A protein expression in Ba/F3 cells. (B) Morphological changes in Ba/F3 cells retrovirally expressing *ALK**,** EML4-ALK, TTC7A-ALK*, an empty vector, or blank control cells, cultured in the presence (+IL3) or absence (-IL3) of interleukin-3 (IL-3) for 5 days. Representative images after 5 days of incubation are shown. (C) Growth curve of Ba/F3 cells carrying *TTC7A-ALK, EML-ALK, ALK*, empty vector and blank cell in the presence of IL-3 (right) and absence of IL-3 (left), respectively. Error bar stands for standard deviation of triplicates. (D) Tumorigenic potential of *TTC7A-ALK* gene fusions in vivo. Tumor growth was significantly promoted by *TTC7A-ALK* and *EML4-ALK*(*V1*) expression and was markedly inhibited after crizotinib treatment. (E) Western blotting and quantifcation analysis of *EML4* and *TTC7A* protein expression in NIH3T3 cells. (F) Activation of downstream signaling pathways by the *TTC7A-ALK* fusion. Data are averages of three independent experiments, and results are representative of two separate replicates. *P < 0.05, **P < 0.01, ***P < 0.001 vs. the Control and NC (negative control) groups. Control, blank control; NC or NEG, negative control; *EML4-ALK* or *EML(V1) -ALK, EML4-ALK* fusion gene.

To investigate whether the novel *TTC7A-ALK* fusion protein contributes to tumorigenesis, we constructed stable expression of *TTC7A-ALK* or *EML4-ALK variant 1* (the most common *EML4-ALK* fusion subtype) in three non-cancer cell lines commonly used to assess carcinogenicity (HEK-293T cells, NIH3T3 fibroblasts and Ba/F3 cells). In Ba/F3 cells, cell proliferation assay demonstrated that *TTC7A-ALK* induced interleukin-3 (IL-3) independent proliferation, while cells carrying the empty vector grew minimally or not at all in the absence of IL-3 (Fig. 4C). Furthermore, the *TTC7A-ALK* group exhibited a greater proliferative capacity compared to the *EML4-ALK(V1)* and wild-type *ALK* groups, both in the presence and absence of IL-3. Microscopic observations showed that the cells expressing *TTC7A-ALK, EML4-ALK(V1)* and *ALK* appeared rounder and denser than those in the blank control group when cultured under the same number and the same culture conditions (Fig. 4B). In xenograft animal models, we found that the overexpression of *TTC7A-ALK* and *EML-ALK (V1)* significantly promoted tumor growth of NIH3T3 cells. After feeding crizotinib, the tumor volumes of *EML4-ALK (V1)* and *TTC7A-ALK* groups were significantly reduced, which indicated that NIH3T3 cells expressing *EML4-ALK (V1)* and *TTC7A-ALK* were highly sensitive to crizotinib treatment in vivo (Fig. 4D). To explore the downstream signaling pathways affected by *TTC7A-ALK*, the fusion protein was transiently expressed in HEK-293T cells.. As shown in Fig. 4F, western blot analysis revealed that, in the presence of *EML4-ALK* and *TTC7A-ALK,* the phosphorylation of ERK1/2 in the Ras/MAPK pathway and AKT (T308) in the PI3K/AKT pathway were increased. Notably, these phosphorylation events were suppressed following crizotinib treatment. These findings indicate that *TTC7A-ALK* induces hyperactivation of the mitogen-activated protein kinase (MAPK) and phosphoinositide 3-kinase/AKT (PI3K/AKT) pathways, contributing to tumor progression.

Together, these results suggest that *TTC7A-ALK* is a potent oncogenic driver capable of promoting IL-3-independent proliferation, tumor growth, and activation of key signaling pathways, and it remains sensitive to crizotinib inhibition.

### *TTC7A-ALK* represents a potential therapeutic target

According to the recommendations for *ALK*-positive advanced NSCLC in the 2019 NCCN guidelines, the patient began oral treatment with crizotinib (250 mg twice daily) in November 2019 immediately after the identification of the *TTC7A-ALK* fusion gene. Crizotinib, a multi-target tyrosine kinase inhibitor (TKI) with activity against *MET, ALK*, and *ROS1*, was approved by the US FDA in 2011 as a first-line therapy for metastatic *ALK*-positive NSCLC [62]. After six months of crizotinib treatment, the patient exhibited a significant clinical and biochemical response. Tumor marker levels, including CEA, decreased dramatically from 506.63 ng/mL before treatment to 23.86 ng/mL (Fig. 1B). These changes were accompanied by marked improvement in clinical symptoms. Meanwhile, A CT scan of chest and abdomen showed a remarkable shrinkage of the tumor in the right hilar region and metastatic lesions in the liver, head of the pancreas, kidney, and right rib (Fig. 1A). According to the Response Evaluation Criteria in Solid Tumors (RECIST) guidelines (version 1.1) [63], the patient achieved partial remission with crizotinib treatment. Treatment was well tolerated, with no severe adverse events reported.

Despite initial success, the patient discontinued regular follow-ups and treatment due to financial constraints. After taking crizotinib for 29 months, the patient returned to the hospital on May 11, 2022, complaining of pain in the waist, back, and left hip, as well as difficulty in moving the left lower limb. Chest and abdominal CT scans revealed increased size and number of lesions in the right hilar region, lung, liver, kidney, ribs, and other metastatic sites compared to previous assessments. Additionally, magnetic resonance imaging (MRI) screening showed multiple bone destructions in the thoracic vertebrae, lumbar vertebrae, pelvis, and left femoral neck, which were considered as metastases. These findings confirmed the development of crizotinib resistance (Supplementary Fig.1), with CEA levels increasing to 1046 ng/mL (see Fig. 1B). We recommended that the patient undergo lung cancer gene testing again to clarify the reason for drug resistance, but the patient's family refused the testing due to financial reasons. According to the recommendations of the guidelines, crizotinib was discontinued, and alectinib was administrated in May 2022. Alectinib treatment resulted in immediate relief of symptoms, a significant reduction in tumor size, and a dramatic decrease in CEA levels to 15.95 ng/mL. This response suggests that *TTC7A-ALK* remained a key driver of the patient' s tumor progression. The patient continues to receive alectinib and remains in stable condition, with the second progression-free survival (PFS) now over 31 months.

## Discussion

With the rapid advancement and widespread application of next-generation DNA sequencing (NGS) technology, an increasing number of *ALK* fusion partners have been identified across various human cancers [[64], [65], [66]]. These *ALK* translocations are known to drive tumorigenesis primarily by enabling constitutive, ligand-independent activation of the *ALK* kinase, often mediated by the fusion partner's ability to promote oligomerization [67]. Among these, *EML4-ALK* is the most common and extensively studied fusion in non-small cell lung cancer (NSCLC). It has demonstrated well-established oncogenic activity and a high degree of sensitivity to *ALK* inhibitors such as crizotinib. However, with the discovery of more rare *ALK* fusion partners, there is limited knowledge regarding their roles in tumorigenesis and their clinical responses to targeted therapies. Investigating these rare fusions is essential for advancing personalized treatment strategies and maximizing therapeutic benefits for NSCLC patients.

In this study, we identified a novel *ALK* rearrangement, *TTC7A-ALK*, in an NSCLC patient using NGS. This fusion results from the inversion involving exons 1–4 of *TTC7A* and exons 1–19 of *ALK*. To the best of our knowledge, this is the first report of *TTC7A* as an *ALK* fusion partner and its role in cancer. The *TTC7A (tetratricopeptide repeat domain 7A)* gene is known to play an intrinsic role in nuclear organization and chromatin structure, influencing transcriptional activity and genome stability. Loss-of-function mutations in *TTC7A* have been implicated in human intestinal and immune disorders [68]. However, its role in oncogenesis has remained unexplored until now. To investigate the oncogenic potential of *TTC7A-ALK* fusion (T4:A19), we introduced it into Ba/F3 cells, NIH3T3 fibroblasts and 293T cells. First, in vitro studies demonstrated that the fusion protein enabled Ba/F3 cells to grow in an IL-3-independent manner, which is a hallmark of oncogenic transformation, and this effect could be inhibited by crizotinib. Then, the results of in vivo experiments further indicated that *TTC7A-ALK* fusion could promote tumorigenesis of NIH3T3 cells in nude mice, and this effect could also be inhibited by crizotinib. Finally, molecular pathway studies in HEK-293T cells indicated that *TTC7A-ALK* induced hyperactivation of the Ras/MAPK and PI3K/AKT signaling pathways, both of which are well-known mediators of tumor progression. and this hyperactivation could also be inhibited by crizotinib treatment. Importantly, crizotinib inhibited the activation of these pathways, highlighting its therapeutic potential against *TTC7A-ALK*-driven tumors. Clinically, the patient harboring this novel fusion responded remarkably well to crizotinib. After the initiation of treatment, the patient experienced significant symptomatic relief, and imaging showed a marked reduction in the size of the tumor. Moreover, the initial progression-free survival (PFS) was extended to 29 months, which is notably longer than the median PFS of 10 months reported in clinical studies of *ALK*-positive NSCLC.

This case underscores the sensitivity of *TTC7A-ALK*-driven tumors to crizotinib, even in the absence of consistent follow-up and monitoring. However, the patient eventually experienced tumor progression and resistance to crizotinib. Further studies need to be conducted to explore the mechanisms of crizotinib resistance in this case.. Due to financial constraints, the patient declined additional NGS testing after disease progression, resulting in a lack of genetic information regarding resistance-associated mutations. It is well-documented that secondary *ALK* mutations, bypass signaling pathways, or phenotypic transformation can contribute to resistance in *ALK*-positive NSCLC. According to the recommendations of the guidelines, crizotinib was discontinued, and alectinib was administrated. Fortunately, after switching to alectinib, a second-generation TKI, the patient exhibited immediate symptom relief, significant lesion control, and a reduction in tumor markers, with the second progression-free survival already exceeding 31 months at the time of this report. The patient's rapid response to alectinib suggests that the relapsed tumor is still *ALK*-driven. However, the mechanism for crizotinib-resistance in this patient is still unknown.

This case highlights several critical insights. First, the novel *TTC7A-ALK* fusion represents a potential therapeutic target that responds well to *ALK* inhibitors such as crizotinib and alectinib. Second, the extended PFS achieved with crizotinib underscores the importance of early and precise molecular diagnosis using NGS to guide targeted therapy. Finally, the study emphasizes the need for continued research to elucidate the mechanisms of resistance in rare *ALK* fusions. Future efforts should focus on overcoming resistance and identifying effective combination therapies to further extend survival and improve outcomes for patients with *ALK*-positive NSCLC.

In conclusion, we suggest that future research could further explore the molecular mechanisms of novel fusions, develop more efficient detection technologies, and expand the sample size to validate the clinical significance of these novel fusions. This would help drive the continuous advancement of detection guidelines and the expansion of their clinical applications.

## Conclusion

In this study, we identified a rare *TTC7A-ALK* fusion in a patient with advanced lung adenocarcinoma who exhibited a prolonged response to crizotinib. Functional studies conducted both in vitro and in vivo demonstrated the oncogenic potential of this fusion gene in Ba/F3 cells and NIH3T3 fibroblasts, with its activity effectively inhibited by crizotinib. The dual findings of the oncogenic role of *TTC7A-ALK* and the patient' s positive clinical response to crizotinib provide valuable new insights into lung cancer therapy. These results underscore the importance of integrating rare fusion gene identification into clinical diagnostics and personalized targeted therapy, potentially improving treatment outcomes for NSCLC patients.

## Informed consent

The patient has provided written informed consent for the release of case details and any accompanying images. This is an observational case report and does not require institutional approval because all treatments the patient received were standard, including *ALK-TKI* (crizotinib).

## CRediT authorship contribution statement

**Meijin Huang:** Writing – review & editing, Writing – original draft, Validation. **Xiangqing Zhu:** Validation, Methodology. **Wenmang Xu:** Validation, Methodology. **Jun Zhu:** Validation, Methodology. **Xin Xun:** Validation, Data curation. **Bin Su:** Validation. **Hong Chen:** Writing – review & editing, Writing – original draft, Conceptualization.

## Declaration of competing interest

The authors declare that they have no known competing financial interests or personal relationships that could have appeared to influence the work reported in this paper.

## Appendix Supplementary materials

Image, image 1

Image, application 2
