# Supplementary figures and images for: TTC7A-ALK, a novel ALK fusion variant identified in a patient with metastatic lung adenocarcinoma, exhibits excellent response to crizotinib
Source: Transl Oncol. 2025 Mar 6;54:102345. doi: 10.1016/j.tranon.2025.102345 (PMC11930134; doi:10.1016/j.tranon.2025.102345)

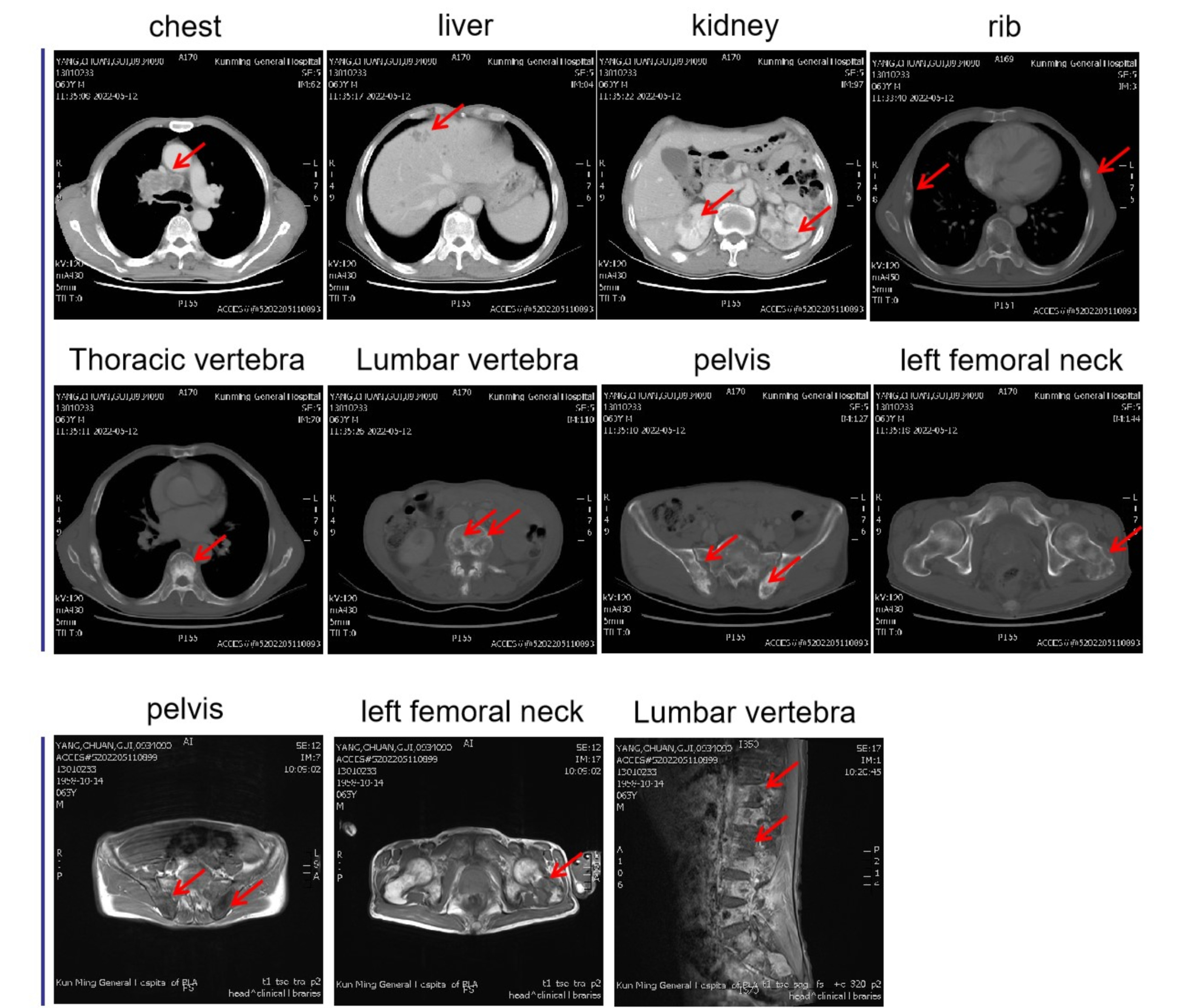

Supplement: Supplementary file 1 [file mmc1.jpg]
